# Urachal mixed adenocarcinoma and small cell neuroendocrine carcinoma with widespread metastasis and resistance to chemotherapy: a case report

**DOI:** 10.1186/s13000-024-01490-5

**Published:** 2024-06-14

**Authors:** Sarah Obiedat, Khaled Murshed, Lajos Szabados, Khaled Al Rumaihi, Issam Al Bozom

**Affiliations:** 1 Department of Laboratory Medicine and Pathology, Hamad Medical Corporation, Doha, Qatar; 2 Department of Radiology, Hamad Medical Corporation, Doha, Qatar; 3 Department of Urology, Hamad Medical Corporation, Doha, Qatar

**Keywords:** Urachus, Adenocarcinoma, Small cell neuroendocrine carcinoma, Metastasis, Chemotherapy

## Abstract

Neuroendocrine carcinoma arising from the urachus is extremely rare. We describe a case of a 33-year-old gentleman who presented with hematuria and diagnosed to have a composite adenocarcinoma and small cell neuroendocrine carcinoma arising from the urachus. The patient also had widespread metastasis at the time of presentation, therefore, he was referred for chemotherapy. However, the disease showed progression despite treatment. Recognition of neuroendocrine carcinoma component in urachal tumors, although rare, is very essential as this histologic type carries poor prognosis with aggressive clinical outcome.

## Introduction

During embryogenesis, a connection forms between the dome of urinary bladder and umbilicus called “the urachus”. After birth, it regresses to form a fibrous remnant called “the median umbilical ligament”. In some individuals, failure of involution of this embryologic structure occurs to form urachal anomalies and urachal remnant. Histologically, this remnant is lined by urothelial mucosa which can undergo metaplastic change to intestinal-type epithelium [1, 2].

Neoplasms arising from the urachus can rarely occur. Adenocarcinoma is the most common histologic type of urachal carcinomas and it comprises 10% of primary bladder adenocarcinomas and less than 1% of all bladder tumors [3, 4]. Urachal carcinomas usually occur in younger individuals with median age of 56 years compared to a median age of 69 years in non-urachal bladder tumors [3, 4].

Although more than two thirds of urachal carcinomas are of adenocarcinoma type, non-glandular urothelial or squamous differentiation comprises 24% of cases [5]. Urachal neuroendocrine carcinoma (NEC) is exceedingly rare, with less than 10 reported cases in the literature [5, 6]. The majority of NECs reported in the literature are composed of small cell neuroendocrine carcinoma with concomitant adenocarcinoma component. Large cell neuroendocrine carcinoma was encountered in two cases [7].

Herein, we document another rare case of mixed adenocarcinoma and small cell neuroendocrine carcinoma arising from the urachus, that presented with widespread distant organ metastasis.

## Case presentation

A 33-year-old man presented to the urology clinic complaining of painless hematuria with clots for the past 4 months. He is a former smoker with no past medical history. A computed topography (CT) scan revealed a urinary bladder mass with external iliac lymphadenopathy and multiple lung nodules. Cystoscopy revealed a nodular tumor measuring 3 to 4 cm located at the bladder dome with calcifications.

**Fig. 1:**
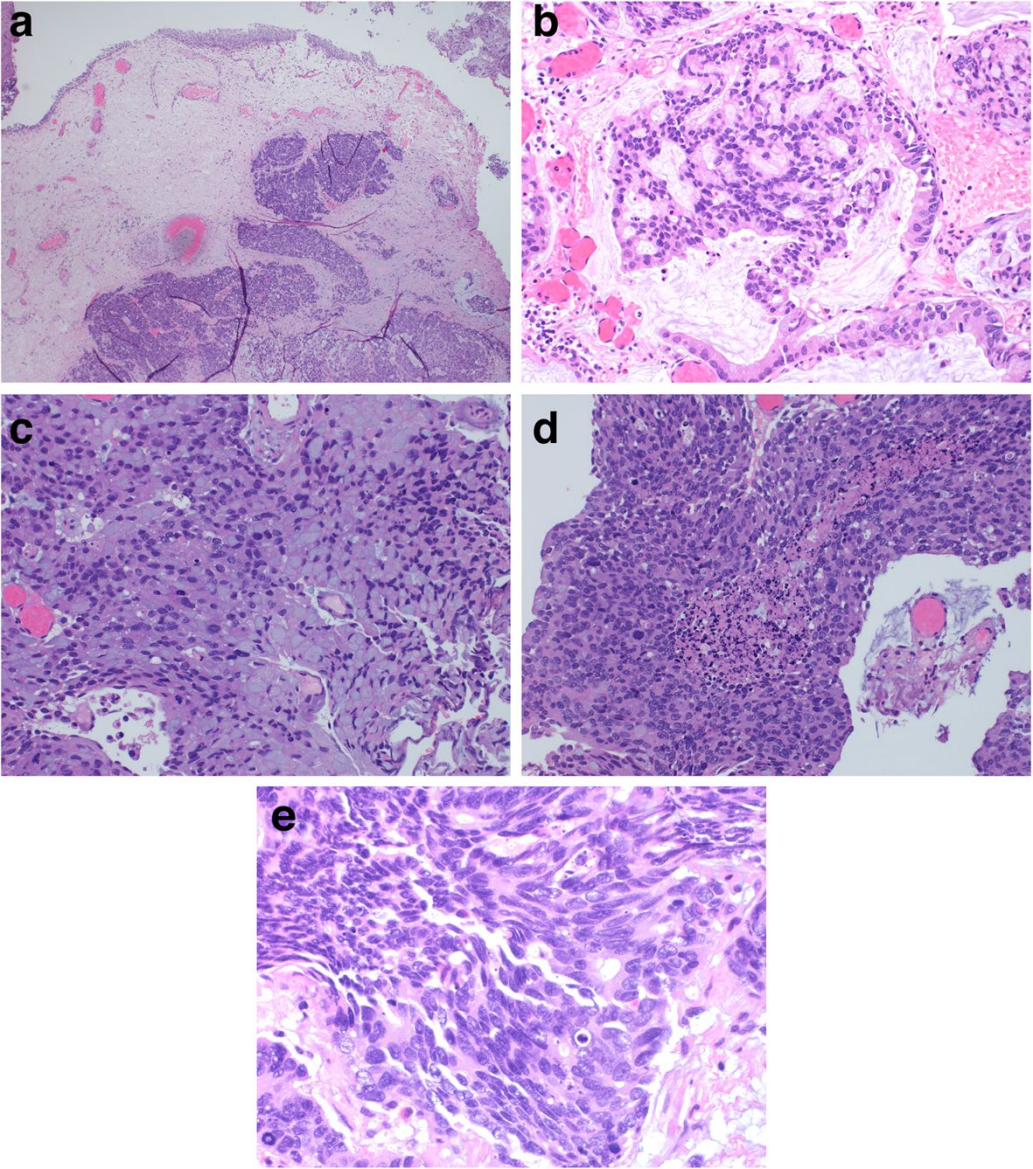
Microscopic features of the tumor. **A**, photomicrograph depicting a tumor located deep in the bladder wall with sharp demarcation from the surface urothelium (Hematoxylin & Eosin stain, × 40). **B**, the adenocarcinoma component of enteric type with extracellular mucin (Hematoxylin & Eosin stain, × 200). **C**, the adenocarcinoma component of signet ring type with intracellular mucin (Hematoxylin & Eosin stain, × 200). **D**, the small cell neuroendocrine carcinoma component with confluent areas of necrosis in the center (Hematoxylin & Eosin stain, × 200). **E**, High-power view of the small cell neuroendocrine carcinoma component with brisk mitosis and apoptosis (Hematoxylin & Eosin stain, × 400)

The patient underwent transurethral resection of bladder tumor (TURBT) for diagnostic and therapeutic purposes. Histopathology showed a tumor with dual components; adenocarcinoma and small cell neuroendocrine carcinoma. The epicenter of the tumor was located in the bladder wall with sharp demarcation from the overlying urothelium (Fig. 1A). The adenocarcinoma component comprised 60% of the whole tumor bulk and was mainly of enteric type with a small component of signet ring type (Fig. 1B and C). In contrast, the neuroendocrine carcinoma component had small cell morphology and composed of hyperchromatic cells with inconspicuous nucleoli, minimal amount of cytoplasm, and brisk mitotic activity, arranged in solid sheets and exhibited areas of confluent necrosis (Fig. 1D and E). The tumor showed extensive invasion into the lamina propria with foci of lymphvascular invasion.

**Fig. 2:**
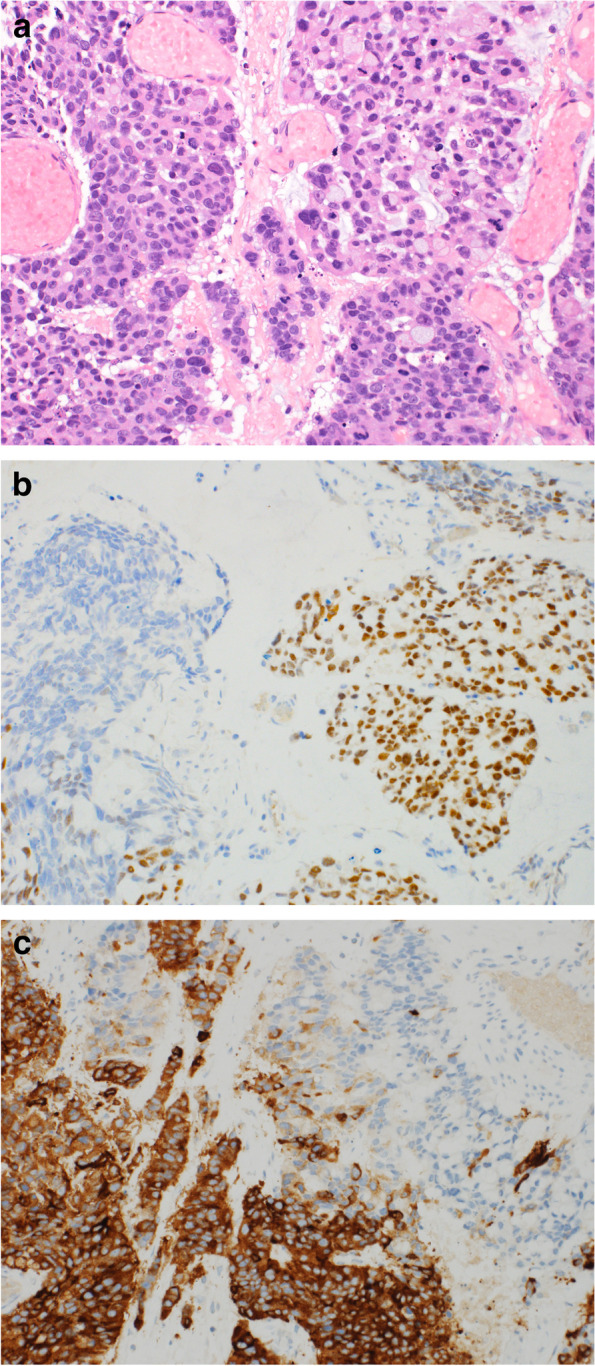
Morphology of tumor with contrasting immunostaining. **A**, Photomicrograph showing adenocarcinoma component with signet ring cells (right) and neuroendocrine carcinoma component (left) (Hematoxylin & Eosin stain, × 200). **B**, positive nuclear staining for CDX2 in the adenocarcinoma component (right) while it’s negative in the neuroendocrine carcinoma component (left). **C**, the small cell neuroendocrine carcinoma component (left) is diffusely and strongly positive for synaptophysin while the adenocarcinoma component (right) is negative

Immunohistochemical studies demonstrated the expression of CDX2 (nuclear) and cytokeratin 20 (cytoplasmic and membranous) in adenocarcinoma component while it was negative for neuroendocrine markers; synaptophysin, chromogranin and CD56. On the contrary, the neuroendocrine carcinoma component was positive for synaptophysin, chromogranin and CD56 (all expressed cytoplasmic staining) (Fig. 2A, B and C). TTF-1 was negative excluding the possibility of lung origin.

**Fig. 3:**
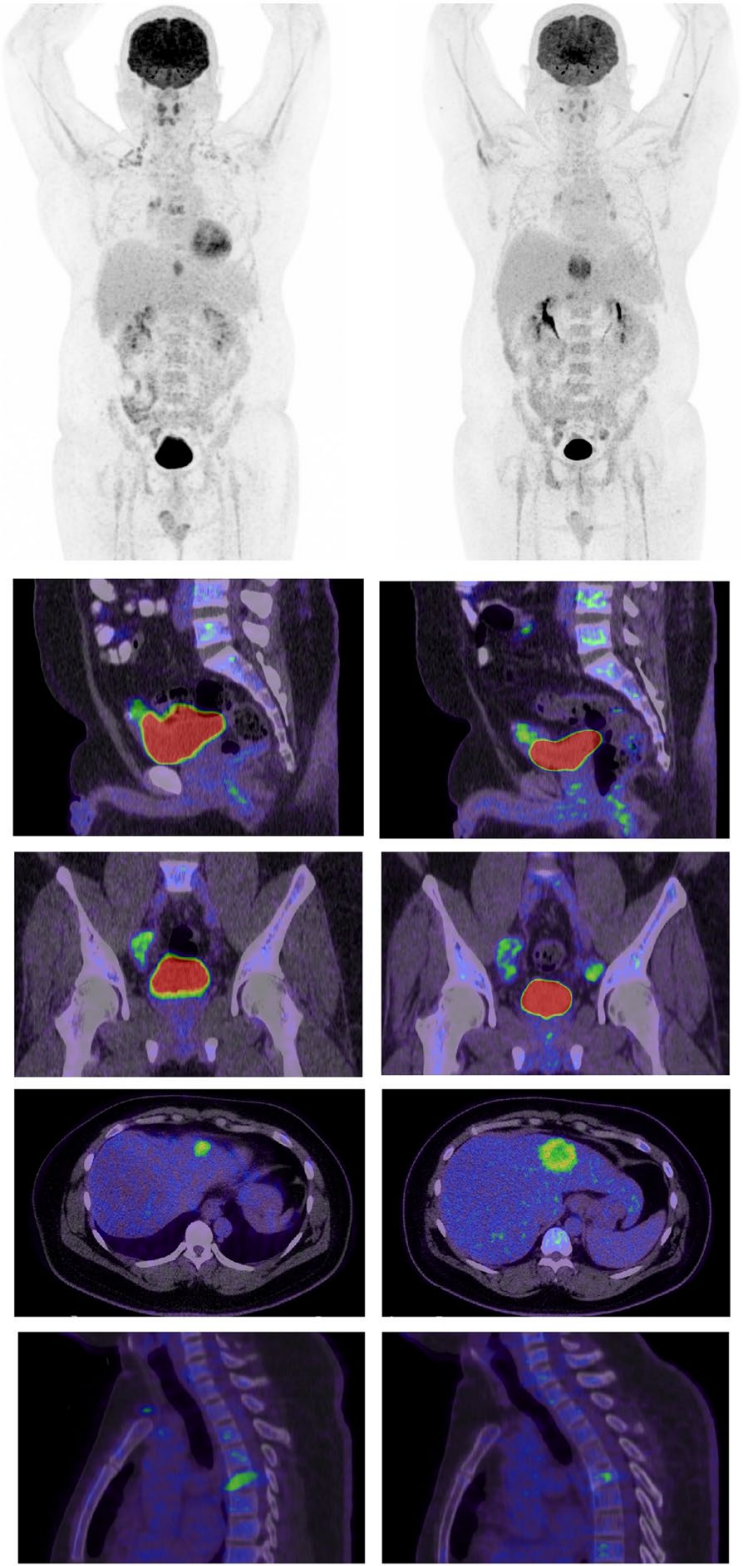
Staging (left column) and restaging (right column) FDG PET/CT scan maximum intensity projection (MIP—**A-B**) and fused sagittal (**C-D** and **I-J**), coronal (**E–F**) and transaxial (**G-H**) images. The primary tumor in the ventral aspect of the urinary bladder (red arrowhead) was seen even with the presence of the excreted FDG in the bladder. Bilateral parailiac lymph nodes showed progression during treatment (blue arrowheads). Liver metastasis in the left lobe has also increased in size (green arrowhead). Irradiated D5 vertebral body lesion partially responded (yellow arrowhead). Physiologic brown fat activity was noted on the staging scan (brown arrowheads)

Following the diagnosis of urachal carcinoma, the patient underwent a full body PET/CT scan which showed hypermetabolic focus at the urinary bladder superior anterior aspect representing a residual tumor (Fig. 3 (A-D)). Multiple hypermetabolic foci at the right iliac and right tracheobronchial lymph nodes as well as vertebral lytic lesions and liver nodule suspicious for metastasis were identified (Fig. 3 (E-J)). Thoracic vertebral body pathological fracture with posterior wall displacement and spinal cord indentation were visualized by dorsal spine MRI.

The patient was labelled as stage IV and was referred for palliative chemotherapy and spine radiotherapy. Three months following diagnosis and starting treatment, MRI lumbar spine with contrast showed no significant change in thoracic vertebral body lesions post radiotherapy. A PET CT scan following two cycles of Carboplatin and Gemcitabine chemotherapy was performed to assist response and showed disease progression with increased uptake in lung and liver lesions, urinary bladder primary mass and newly developed pelvic and retroperitoneal hypermetabolic lymph nodes (Fig. 3). The patient was switched to a second line of palliative chemotherapy of folinic acid, Fluorouracil and oxaliplatin (FOLFOX).

## Discussion

The urachus is a tubular structure that extends from the dome of urinary bladder to the umbilicus during embryogenesis. After birth, it undergoes involution to form the median umbilical ligament. However, it may persist until adulthood to form urachal remnant [1, 2]. Carcinomas can rarely arise from the epithelium that lines the urachal remnant. The most common histological type of carcinoma that arises from urachal remnant is adenocarcinoma. However, non-glandular carcinomas such as urothelial carcinoma, neuroendocrine carcinoma and squamous cell carcinoma are reported. They may occur in pure form, as a minor component in an adenocarcinoma predominant urachal carcinoma or as predominant pattern admixed with focal adenocarcinoma [5].

The initial presentation of urachal carcinoma is indistinguishable from urothelial carcinoma including hematuria, suprapubic pain and voiding disturbances. Diagnostic techniques performed during the diagnosis includes radiographic imaging, cystoscopy, and endoscopic biopsy [8]. CT and MRI imaging modalities yield strong supporting findings. The presence of bladder dome cystic and solid mass with small calcifications is considered pathognomonic of urachal carcinoma. Tumors with this appearance should be considered urachal in origin unless proven otherwise [9]. Serum levels of carcinoembryonic antigen (CEA), CA125, and CA19 –9 are found to be elevated in 40–60% of cases [10].

Urachal neuroendocrine carcinoma (NEC) is very rare with only a handful number of cases reported in the literature. Wang et al. reported the largest series of three cases of urachal NEC [7]. In that study, all the patients were young with median age of 27-year-old and the male to female ratio was 2 to 1. All three cases had an adenocarcinoma component admixed with NEC. Two of the three cases showed small cell NEC component while one case had large cell NEC component. Paner et al. reported seven cases of non-glandular urachal carcinoma, two of them were pure small cell NEC [5]. In our case, there was an adenocarcinoma component of enteric type with focal signet ring changes admixed with NEC component that showed small cell morphology.

Primary non-urachal adenocarcinoma with neuroendocrine differentiation of the urinary bladder should be considered in the differential diagnosis. The presence of cystitis glandularis and carcinoma in situ elements would favor the diagnosis of non-urachal adenocarcinoma. In addition, the location of tumor at bladder dome with its epicenter in bladder wall are features that favor urachal origin [11]. In our case, there was no cystitis glandularis or carcinoma in situ components. The tumor was located in the bladder dome and had an intramural epicenter with sharp demarcation from the surface epithelium. These findings are in keeping with urachal origin. Metastatic NEC should also be considered. However, the patient in our case did not have primary tumors elsewhere by imaging.

Non-glandular urachal carcinoma prognosis, in pure form or as mixed tumors, appears to be very poor. In one series, six out of seven patients developed extension outside urachus and bladder in addition to metastasis, and all 6 died of disease within 2 years. Two of these patients had pure small cell carcinoma, two with pure urothelial carcinoma and the remaining three had urothelial carcinoma with squamous differentiation, urothelial carcinoma with focal signet ring cells and one with focal glandular differentiation [12].

NEC of the urachus appears to have the most aggressive clinical behavior. All three cases reported by Wang et al. had widespread metastases [7]. In addition, the two cases of small cell NEC reported by Paner et both had metastasis to the lymph nodes and bone [5]. The behavior of tumor in our case was similar to the cases reported in literature as the patient had widespread distant metastasis to the liver, iliac and tracheobronchial lymph nodes, thoracic vertebra and lung.

Due to the aggressive behavior of urachal carcinoma, the main therapeutic strategy is a combination of urachal ligament and umbilicus en block surgical excision with radical or partial cystectomy and pelvic lymphadenectomy. Adjuvant chemotherapeutic agents are used in most cases, although no standard regimen is yet available, cisplatin-based combination therapies (gemcitabine) and 5-Flouruoracil (FU) are most commonly used with a response rate of 30–40%. Nevertheless, long term survival rate continues to be low [13]. One study reported the successful treatment of a patient with urachal carcinoma by using the immunosuppressant atezolizumab, an anti-PDL-1 antibody [14]. In our case, the patient was not offered immunotherapy because PDL-1 immunohistochemical staining was negative with a CPS score of < 1.

## Conclusion

We describe a rare case of composite adenocarcinoma and small cell NEC of urachal origin with widespread metastasis not responding to treatment. Although rare, the recognition of NEC component in urachal carcinoma is very crucial as it carries poor prognosis and affects the treatment modalities and chemotherapeutic regimens that the patient would receive.

## Data Availability

The data that support the findings of this study are available from the corresponding author upon reasonable request.
